# Involved field radiotherapy (IFRT) versus elective nodal irradiation (ENI) for locally advanced non-small cell lung cancer: a meta-analysis of incidence of elective nodal failure (ENF)

**DOI:** 10.1186/s13014-016-0698-3

**Published:** 2016-09-21

**Authors:** Ruijian Li, Liang Yu, Sixiang Lin, Lina Wang, Xin Dong, Lingxia Yu, Weiyi Li, Baosheng Li

**Affiliations:** 1 Department of Radiation Oncology, Shandong Cancer Hospital and Institute Affiliated to Shandong University, Provincial Key Laboratory of Radiation Oncology, Jiyan Road 440, 250117 Jinan, Shandong China; 2 Department of Radiation Oncology II, Yantai Affiliated Hospital of Binzhou Medical University, Key Department of Yantai Health Bureau, Yantai, Shandong China

**Keywords:** Non-small cell lung cancer, Involved field radiotherapy, Elective nodal irradiation, Elective nodal failure

## Abstract

**Background and purpose:**

The use of involved field radiotherapy (IFRT) has generated concern about the increasing incidence of elective nodal failure (ENF) in contrast to elective nodal irradiation (ENI). This meta-analysis aimed to provide more reliable and up-to-date evidence on the incidence of ENF between IFRT and ENI.

**Materials and methods:**

We searched three databases for eligible studies where locally advanced non-small cell lung cancer (NSCLC) patients received IFRT or ENI. Outcome of interest was the incidence of ENF. The fixed-effects model was used to pool outcomes across the studies.

**Results:**

There were 3 RCTs and 3 cohort studies included with low risk of bias. There was no significant difference in incidence of ENF between IFRT and ENI either among RCTs (RR = 1.38, 95 % CI: 0.59–3.25, *p* = 0.46) or among cohort studies (RR = 0.99, 95 % CI: 0.46–2.10, *p* = 0.97). There was also no significant difference in incidence of ENF between IFRT and ENI when RCTs and cohort studies were combined (RR = 1.15, 95 % CI: 0.65–2.01, *p* = 0.64). *I*^2^ of test for heterogeneity was 0 %.

**Conclusion:**

This meta-analysis provides more reliable and stable evidence that there is no significant difference in incidence of ENF between IFRT and ENI.

**Electronic supplementary material:**

The online version of this article (doi:10.1186/s13014-016-0698-3) contains supplementary material, which is available to authorized users.

## Introduction

Combination of chemotherapy and external beam radiotherapy is the standard treatment for locally advanced non-small cell lung cancer (NSCLC) patients. Traditionally, external beam radiotherapy for patients with locally advanced NSCLC targets the primary tumor as well as the ipsilateral hilar and mediastinal nodal stations, and sometimes the supraclavicular fossa nodal stations, even if there is no evidence of clinical involvement of all nodal stations. This technique is known as elective nodal irradiation (ENI). However, ENI will limit dose escalation because of pulmonary and esophageal toxicities, and lower radiation dose is unfavorable to local tumor control [1]. This calls for the omission of ENI and use of another radiation treatment technique, which is called involved field radiotherapy (IFRT). IFRT allows higher radiation dose to the primary tumor with the goal of reducing local failure.

However, the trend of using IFRT has generated concern about the increasing incidence of nodal failure in untreated nodal stations. Some studies reported the crude incidences of elective nodal failure (ENF) were below 10 % when IFRT were applied [2–4], particularly in positron emission tomography-computed tomography (PET-CT) staged patients [5, 6], while existing evidences for this were weak until now. Now the National Comprehensive Cancer Network (NCCN) guidelines recommend IFRT omitting ENI is category 2A [7]. We, therefore, carried out a systematic review and meta-analysis to provide more reliable and up-to-date evidence on the incidence of ENF between IFRT and ENI and to identify whether the IFRT was as safe as reported.

## Materials and methods

### Study design

This was a systematic review carried out in accordance with the Cochrane Collaboration Handbook for Systematic Reviews of Interventions [8]. The manuscript was arranged according to the Preferred Reporting Items for Systematic Reviews and Meta-Analysis (PRISMA) statement [9].

### Search strategy

In order to achieve the maximum sensitivity, two review authors (RJL and LY) independently searched through the PubMed (1966 to February 2016), Embase (1988 to February 2016), and Cochrane Central Register of Controlled Trials (February 2016, Issue 2) databases to find relevant articles using the following search strategy: (“Non-small cell lung cancer” [all fields]) AND ( (“Involved field radiotherapy” [all fields] OR “Involved field irradiation” [all fields]) OR (“Elective nodal irradiation” [all fields] OR “Selective nodal irradiation” [all fields])). The reference lists of relevant articles were further explored manually.

### Inclusion and exclusion criteria

According to the purpose of this meta-analysis, ENF was defined as an uninvolved nodal failure without local failure, that is, any lymph nodes of failure in region only got prophylactic irradiation in the ENI and corresponding region initially uninvolved in the IFRT. Failure in the uninvolved lymph nodes that occurred with distant metastases without local failure was also considered as ENF.

Only studies that investigated the locally advanced NSCLC patients who received 3-dimensional conformal or intensity modulated radiotherapy (concurrent chemotherapy, sequential chemotherapy, or not) with the use of IFRT or ENI were eligible for inclusion in our meta-analysis. Only the photon therapy was allowed. Studies with no ENF data available, with single treatment arm and containing less than 20 patients in each treatment arm were excluded. Expert opinions, reviews, and letters were excluded in case of publication bias. Besides, the studies were limited to English publications in humans. All the articles were filtered by inclusion and exclusion criteria.

### Selection of studies and data collection

Three reviewers (SXL, XD and LXY) independently assessed the eligibility of abstracts identified by the search. The full article that appeared to meet the inclusion criteria was retrieved for closer examination. Disagreement over eligibility of a study was resolved by consensus. The meta-analysis was performed to compare IFRT with ENI by estimating the risk ratio (RR) of ENF. Our primary outcome was the incidence of ENF. For each study, we extracted the key information as following: first author’s name, year of publication, number of patients, regimens for intervention and control arms, duration of follow up, as well as the incidence of ENF. According to the inclusion criteria, only the data of locally advanced NSCLC patients were eligible for extraction. If the data extraction of locally advanced was unable to be finished, the study was excluded.

### Assessment of the risk of bias in included studies

Two reviewers (LNW and WYL) used the Cochrane risk of bias table [8] to randomized controlled trial (RCT) and the Newcastle-Ottawa Scale [10] to cohort study to assess the risk of bias independently. Six domains were employed in the Cochrane Collaboration guidelines including random sequence generation, allocation concealment, blinding of participants or outcome assessment, incomplete outcome data, selective outcome reporting, and other sources of bias. The Newcastle-Ottawa Scale assigned a maximum score of 4 for selection, 2 for comparability, and 3 for outcome. The quality score was ranked as low (≤5 points) or high (≥6 points). As a result, studies ranked as low quality level will be excluded. If necessary, a third reviewer (BSL) would solve disagreements.

### Statistical analysis

We calculated the RR for dichotomous data with proper algorithm. RR and 95 % confidence interval (CI) were calculated for each study in an intent-to-treat analysis. Heterogeneity across studies was assessed with a forest plot and the inconsistency statistic (I^2^). If the heterogeneity was moderate or severe (I^2^ ≥ 50 %), a random-effects analysis model would be applied; otherwise, the fixed-effects analysis model would be applied. All calculations were performed using Review Manager (version 5.3, the Cochrane Collaboration). A 2-sided *p* < 0.05 was considered statistically significant. Graphical funnel plot was generated to visually inspect for publication bias.

## Results

### Search results and characteristics of the included studies

**Fig. 1:**
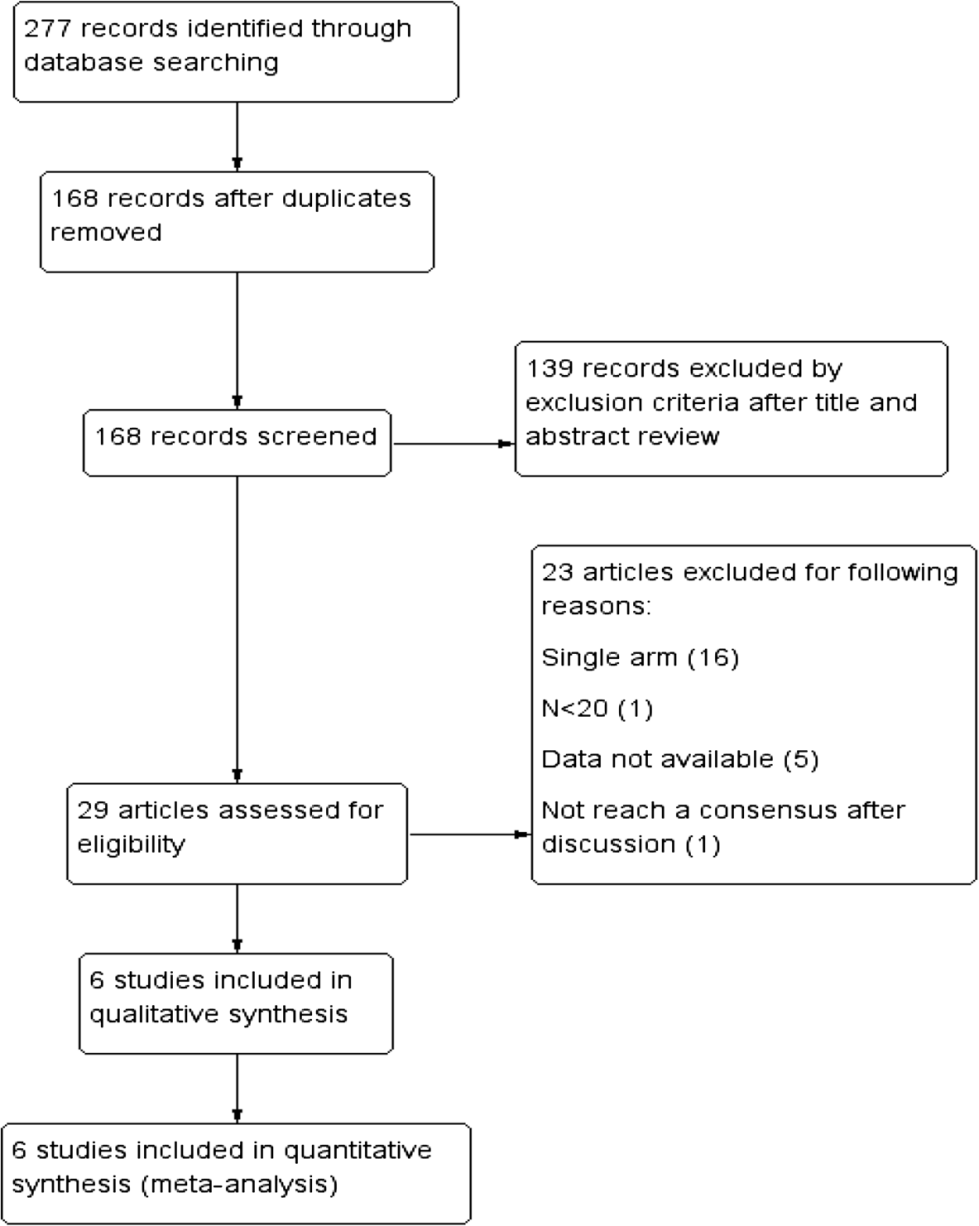
Selection and evaluation process of the eligible studies

A total of 277 potential articles were identified by the literature search; of these articles, 248 were filtered out using our exclusion criteria after screening the titles and abstracts. 29 studies were selected for further review. After the intensive assessment and group discussion, 6 studies [11–16] were chosen for data extraction and meta-analysis (Fig. 1). 3 of 6 studies were RCTs [11–13], and the remaining three were cohort studies [14–16]. One study [15] included stage I-III patients, and only the data of stage III patients were eligible for extraction according to the inclusion criteria. Furthermore, all the patients of this study underwent PET-CT based radiotherapy planning, which was not mandatory in other studies. One study [14] included 10 stage IV oligometastatic patients, but their oligometastatic lesions were treated definitively, which had no effect on ENF. The characteristics of these 6 studies were shown in Table 1.

**Table 1:** Characteristics of included studies

Study	Years of inclusion	Study arms	Patients (n)	RT dose	Chemotherapy	Follow-up time (months)	Number of ENF
RCTs							
Yuan 2007	1997–2001	IFRT	100	68-74Gy	Concurrent	27 (median)	7
		ENI	100	60-64Gy		27 (median)	4
Yang 2007	2002–2004	IFRT	29	66-74Gy	Sequential	Till December 2005	3
		ENI	26	56-70Gy		Till December 2005	1
Chen 2013	2002–2011	IFRT	45	38-74Gy	Concurrent	14.1 (median)	2
		ENI	54	32-70Gy		14.1 (median)	4
Cohort studies							
Fernandes 2010	2003–2008	IFRT	48	60-84Gy	Concurrent or sequential	13.5 (median)	6
		ENI	60	54-72Gy		17 (median)	7
Kolodziejczyk 2012	2008–2009	IFRT	35	58.8Gy	Sequential	32 (median)	1
		ENI	17	58.8Gy		32 (median)	0
Topkan 2015	2007–2012	IFRT	143	60–66Gy	Concurrent	23.3 (median)	3
		ENI	844	60–66Gy		23.3 (median)	21

Note: *IFRT* involved field radiotherapy, *ENI* elective nodal irradiation, *ENF* elective nodal failure, *Gy* gray

### Methodological quality of studies

All the 3 RCTs were classified as unclear risk of bias taking into account the lacking details of blinding of participants and allocation concealment according to the Cochrane risk of bias table. However, except for these items, 3 RCTs were considered as low risk of bias. Based on the Newcastle-Ottawa Scale to assess the risk of bias of the 3 cohort studies, all of them were rated as a total score of ≥7 points, which indicated a low risk of bias. The methodological quality of the included studies was presented in Additional files 1 and 2.

### Meta-analysis of incidence of ENF

**Fig. 2:**
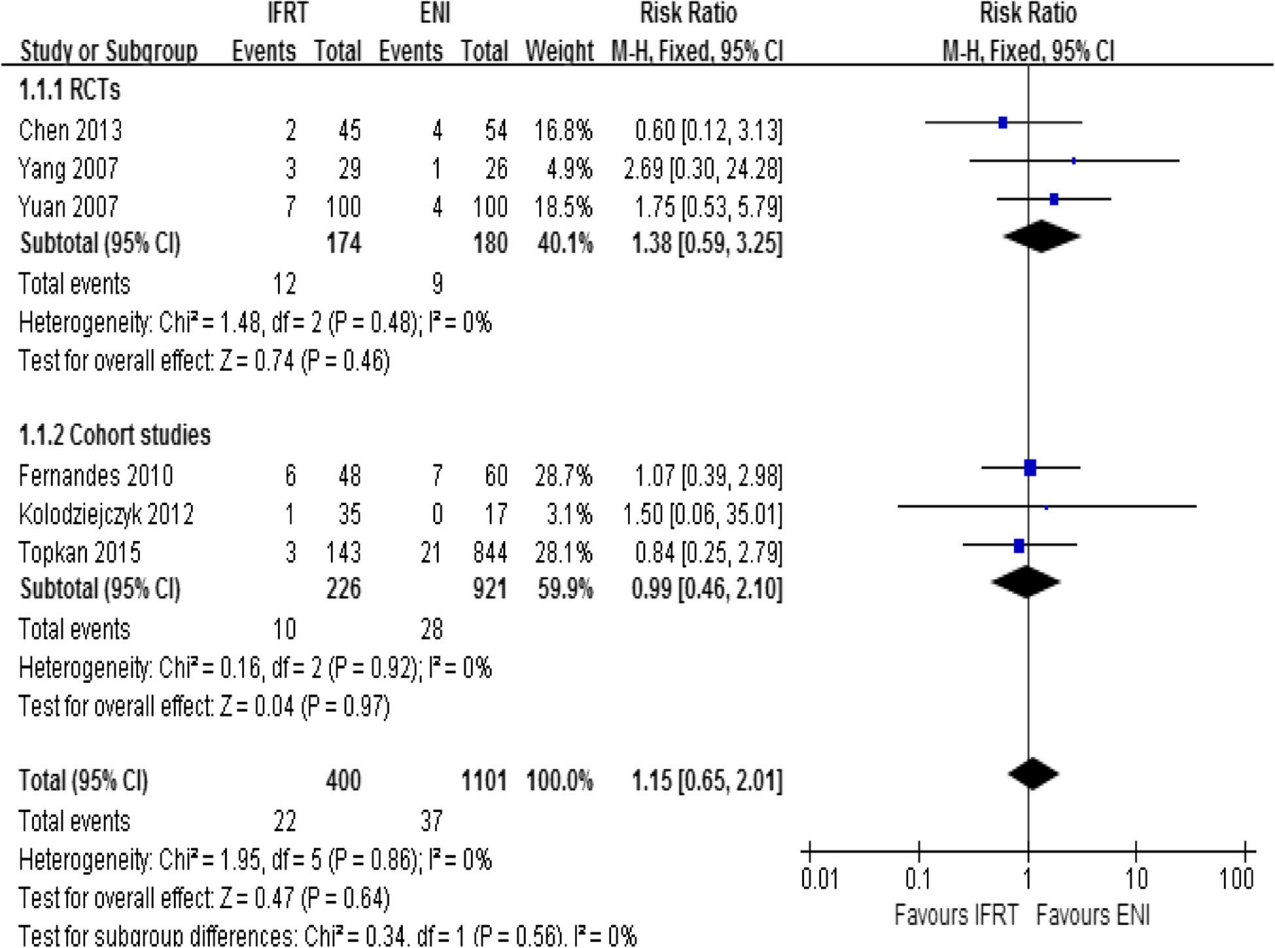
Meta-analysis of incidence of ENF between IFRT and ENI

There were 3 RCTs and 3 cohort studies in the meta-analysis. In RCTs, a total of 354 patients were assigned for IFRT group (*n* = 174) versus ENI group (*n* = 180), and the overall incidence of ENF was 6.9 % in the IFRT group (12 incident cases) and 5.0 % in the ENI group (9 incident cases). In cohort studies, 1147 patients were assigned for IFRT group (*n* = 226) versus ENI group (*n* = 921), and the overall incidence of ENF was 4.4 % in the IFRT group (10 incident cases) and 3.0 % in the ENI group (28 incident cases). There was no significant difference in incidence of ENF between IFRT and ENI either among RCTs (RR = 1.38, 95 % CI: 0.59–3.25, *p* = 0.46; Fig. 2) or among cohort studies (RR = 0.99, 95 % CI: 0.46–2.10, *p* = 0.97; Fig. 2) assuming a fixed-effects model. When combining RCTs with cohort studies, a total of 1501 patients were assigned for IFRT group (*n* = 400) versus ENI group (*n* = 1101), and the overall incidence of ENF was 5.5 % in the IFRT group (22 incident cases) and 3.4 % in the ENI group (37 incident cases). There was no significant difference in incidence of ENF between IFRT and ENI assuming a fixed-effects model (RR = 1.15, 95 % CI: 0.65–2.01, *p* = 0.64; Fig. 2). Furthermore, there were no evidence of heterogeneity among 3 RCTs (Chi^2^ = 1.48, *p* = 0.48, *I*^2^ = 0 %; Fig. 2), 3 cohort studies (Chi^2^ = 0.16, *p* = 0.92, *I*^2^ = 0 %; Fig. 2), and all the included studies (Chi^2^ = 1.95, *p* = 0.86, *I*^2^ = 0 %; Fig. 2).

## Publication bias

**Fig. 3:**
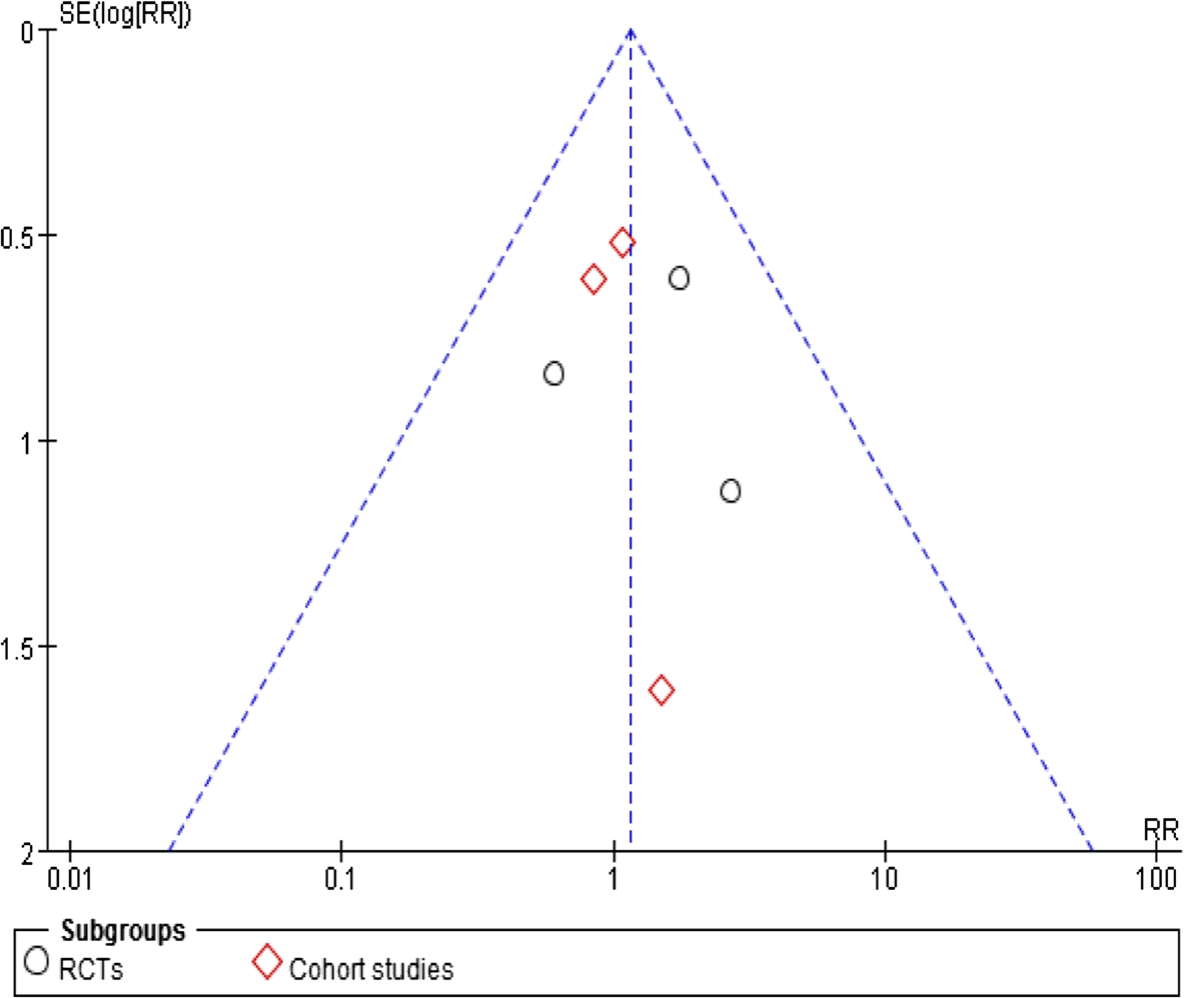
Funnel plot of included studies for primary outcome

We had applied sensitive search strategies and rigorous inclusion criteria to minimize the potential publication bias. According to the funnel plot, no significant asymmetry was detected for our outcome (Fig. 3).

## Discussion

Radiotherapy with ENI has been used since the 2-dimensional radiotherapy era for the improvement of patient’s local control and survival. But emerging evidences from RTOG trials showed that elective irradiation of mediastinal, contralateral hilar and supraclavicular lymph nodes may not be necessary in the treatment of unresectable NSCLC [17]. In addition, the rate of ENF was really low in clinical practice without ENI [2–6]. Thus, more and more treatment guidelines [7, 18] do not recommend the use of ENI in NSCLC. We believe that it may be attributed to the following reasons: (1) The development of 3-dimensional conformal and intensity modulated radiotherapy techniques enable dose escalation especially in use of IFRT, and dose escalation is no doubt favorable to local control [19, 20]. Moreover, IFRT can reduce toxicity by virtue of a decrease in radiation volume. (2) Higher sensitivity of imaging for involved lymph nodes, especially the use of PET-CT [21], which is supposed to lead to better radiation volume tailoring [22]. (3) Concurrent or sequential use of chemotherapy with radiotherapy may also help control local diseases. (4) Although the elective nodal regions are not included in IFRT, they are incidentally irradiated with prophylactic doses, and the prophylactic doses may contribute to the low incidence of ENF [23–26]. Before this meta-analysis, despite the widespread abandoning of ENI for NSCLC patients, we still take a conservative approach in our department because existing evidence for this is insufficient. It consists of only three small RCTs and three cohort studies. We believe that meta-analysis can serve as a valuable tool for studying rare and unintended effects of a treatment, and it can extend prior randomized and nonrandomized studies by permitting synthesis of data and providing more stable estimate of effect. Based on the above, we decided to carry out this meta-analysis.

In this meta-analysis, there were three RCTs and three cohort studies. There was no significant difference in incidence of ENF between IFRT and ENI either among RCTs (RR = 1.38, 95 % CI: 0.59–3.25, *p* = 0.46) or among cohort studies (RR = 0.99, 95 % CI: 0.46–2.10, *p* = 0.97) assuming a fixed-effects model. The Cochran’s Q test resulted in a *p* = 0.48 and a *p* = 0.92 respectively, and the quantity *I*^2^ both were 0 %, indicating that the studies were homogeneous. So we performed a combined analysis of RCTs and cohort studies, and there was also no significant difference in incidence of ENF between IFRT and ENI assuming a fixed-effects model (RR = 1.15, 95 % CI: 0.65–2.01, *p* = 0.64). The quantity *I*^2^ was also 0 %, indicating that there was no evidence of heterogeneity among all the included studies.

Our meta-analysis demonstrated that the difference in incidence of ENF was not significant between IFRT and ENI. This meta-analysis had two main strengths. Firstly, it summarized the highest quality data available comparing IFRT with ENI for locally advanced NSCLC patients until now. Secondly, there was no heterogeneity in the results of the included studies. Nevertheless, this meta-analysis had several limitations. Firstly, the quality of the data was different. For example, this meta-analysis included three cohort studies, which lacked the experimental random allocation of the intervention in contrast to RCTs. Secondly, we compared outcomes across studies but not within studies, so the balance of baseline characteristics between the treatment groups might be neglected. For example, the IFRT group received higher radiation doses than the ENI group in most studies, and statistical analysis showed that the difference was significant in some studies [12, 14]. Thirdly, the follow-up time was relatively short in some studies [13], and some patients might have not suffered from ENF or have died before they suffered from ENF.

## Conclusion

To the best of our knowledge, this is the first meta-analysis of published studies to evaluate the difference in incidence of ENF between IFRT and ENI. It provides more reliable and stable evidence that there is no significant difference in incidence of ENF between IFRT and ENI. IFRT will inevitably take the place of ENI.

## Additional files

Additional file 1: Figure S1. Quality assessment of RCTs using the Cochrane risk of bias table. (DOC 28 kb) Additional file 2: Table S1. Quality assessment of cohort studies using the Newcastle–Ottawa scale. (DOC 26 kb)

## Abbreviations

ENF: Elective nodal failure
ENI: Elective nodal irradiation
IFRT: Involved field radiotherapy
