# Supplementary material for: Involved field radiotherapy (IFRT) versus elective nodal irradiation (ENI) for locally advanced non-small cell lung cancer: a meta-analysis of incidence of elective nodal failure (ENF)
Source: Radiat Oncol. 2016 Sep 21;11:124. doi: 10.1186/s13014-016-0698-3 (PMC5031253; doi:10.1186/s13014-016-0698-3)
Supplement: Additional file 1: Figure S1. — Quality assessment of RCTs using the Cochrane risk of bias table. (DOC 28 kb) [file 13014_2016_698_MOESM1_ESM.doc]

Fig. 4. Quality assessment of RCTs using the Cochrane risk of bias table.
