# Supplementary material for: Involved field radiotherapy (IFRT) versus elective nodal irradiation (ENI) for locally advanced non-small cell lung cancer: a meta-analysis of incidence of elective nodal failure (ENF)
Source: Radiat Oncol. 2016 Sep 21;11:124. doi: 10.1186/s13014-016-0698-3 (PMC5031253; doi:10.1186/s13014-016-0698-3)
Supplement: Additional file 2: Table S1. — Quality assessment of cohort studies using the Newcastle–Ottawa scale. (DOC 26 kb) [file 13014_2016_698_MOESM2_ESM.doc]

Table 2. Quality assessment of cohort studies using the Newcastle–Ottawa scale.

| Study author and year Selection Comparability Outcome Total Scores |
| --- |
| Fernandes 2010 4 2 2 8  Kolodziejczyk 2012 4 0 3 7  Topkan 2015 4 2 3 9 |
